# Myco- and microbiological profiling of a human cadaver reveals drug-resistant strains and new fungal records

**DOI:** 10.1007/s00253-025-13654-4

**Published:** 2025-12-11

**Authors:** Klaudyna Spychała, Agata Piecuch, Kamila Korzekwa, Łukasz Szleszkowski, Agata Thannhäuser, Jędrzej Siuta, Marcin Kadej, Rafał Ogórek

**Affiliations:** 1 Department of Mycology and Genetics, Faculty of Biological Sciences, University of Wrocław, Przybyszewskiego 63, PL-51-148 Wroclaw, Poland; 2 Department of Microbiology, Faculty of Biological Sciences, University of Wrocław, Przybyszewskiego 63, PL-51-148 Wroclaw, Poland; 3 Department of Forensic Medicine, Wroclaw Medical University, Mikulicza-Radeckiego 4, PL-50-345 Wrocław, Poland; 4 Centre for Forensic Biology and Entomology, Department of Invertebrate Biology, Evolution and Conservation, Faculty of Biological Sciences, University of Wrocław, Przybyszewskiego 65, PL-51-148 Wrocław, Poland

**Keywords:** Fungi, Bacteria, Cadaver, Outdoor, Pathogenicity, Active decay

## Abstract

**Abstract:**

In this study, the composition of the postmortem mycobiome and microbiome of a cadaver in an advanced stage of decomposition, had been deposited outdoors and showed extensive mycelial growth, was characterized using culture methods. This approach allowed for the identification of a total of 26 fungal and 16 bacterial species. The dominant fungal species were *Penicillium polonicum*, *Debaryomyces hansenii*, and *Penicillium commune*. Sensitivity tests for voriconazole and amphotericin B were also performed, to which several isolates were resistant. In the case of bacteria, the distribution of dominant species differed between samples taken from outside the body and samples taken from inside the body. Sensitivity tests for 16 antibiotics showed that 23.08% of isolates were resistant to the tested drugs. Importantly, to the best of our knowledge, we detected several species that have not been previously associated with cadavers: *Botryotrichum domesticum*, *Chaetomium subaffine*, *Penicillium allii*, *Scopulariopsis crassa*, *Scopulariopsis fusca* and *Yarrowia deformans*. These results not only expand our understanding of the ecological roles of fungi in cadaver decomposition but also highlight their potential forensic value. First and foremost, it broadens our understanding of local fungal communities associated with human remains, which in the future may provide valuable information about the location or environmental conditions of body deposition, while specific taxa could assist in estimating the postmortem interval. Moreover, the identification of drug-resistant strains underscores the importance of biosafety in forensic practice and raises awareness of the potential for pathogen dissemination from decomposing remains.

**Key points:**

• *Twenty-six fungi and sixteen bacterial species were identified from the cadaver*

• *New fungal records isolated from human remains, expanding forensic mycology knowledge*

• *Antimicrobial susceptibility testing revealed the presence of drug-resistant fungal and bacterial isolates*

**Graphical Abstract:**

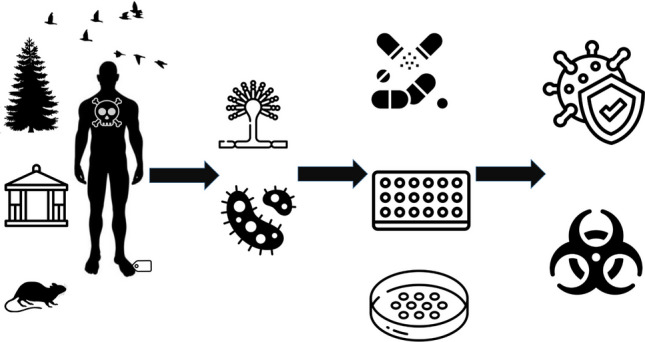

## Introduction

Forensic mycology and microbiology are still developing fields of forensic sciences. Identification of microorganisms on decomposing corpses may be helpful in various aspects, with the estimation of the post mortem interval (PMI) being most widely investigated (Zhou and Bian 2018). This, however, can be hindered by multiple factors related to the environmental conditions and deceased individual features (e.g., health condition, life style) (Tozzo et al. 2022). Identification of post-mortem microbial communities may also provide insights into the ante-mortem health state by detecting potential pathogens. It is postulated that the postmortem microbiome, which during the first 24–48 h after death represents the microbiome of the living host, and specific indicators of the postmortem microbiota (such as specific bacterial taxa) may be an important predictor of heart disease (Pechal et al. 2018). Thus, it is important to expand the knowledge of the post-mortem micro- and mycobiome.

During the decomposition of a body, the microbial community undergoes several significant transitions that correspond to distinct stages of the process. The microbes present in the early phases differ markedly from those found in the later stages (Metcalf et al. 2013). Typically, decomposition progresses through five stages: fresh, bloat, active decay, advanced decay, and dry remains (Payne 1965). The initial stage of decomposition is mainly driven by the activity of intestinal microorganisms (Metcalf et al. 2013). However, the active stage of corpse decomposition usually begins with a change to an aerobic environment (Javan et al. 2019). This leads to a decline in the number of anaerobic species and a dominance of aerobic microorganisms, including those of environmental origin (Speruda et al. 2022). The advanced decomposition stage, continues until all or most of the soft tissue has been broken down and the bones are exposed (Clark et al. 1997). Postmortem autolysis of human cells creates a nutrient-rich environment that supports the proliferation of microorganisms (Cláudia-Ferreira et al. 2023). Furthermore, the cessation of immune system activity permits the expansion of microbial communities originating both endogenously and exogenously (Cláudia-Ferreira et al. 2023). Studies have demonstrated that gut bacteria can translocate to extraintestinal sites as early as five minutes after death (Heimesaat et al. 2012). In contrast, fungal growth becomes macroscopically visible on the surface of the body after several days, depending on environmental conditions (Spychała et al. 2024).

According to Sidrim et al. (2010), outdoor cadaver deposition sites promote fungal proliferation. In the putrefaction stage, the dominant genera in their study were *Aspergillus*, *Penicillium*, and *Candida*. Furthermore, mycological analyses conducted by Sidrim et al. (2010) and Schwarz et al. (2015) indicate that the active decay stage is characterized by the highest fungal species diversity. This conclusion is supported by Gemmellaro et al. (2023), who reported a significant increase in fungal richness as decomposition progresses through the bloating, decay, and active decay stages. One possible explanation for this diversity is the increased availability of substrates utilized by fungi during these phases (Gemmellaro et al. 2023).

Microorganisms—especially bacteria—play a pivotal role in the decomposition of human cadavers, a phenomenon that is receiving growing attention within the scientific community (Nodari et al. 2024). Current research increasingly explores the potential application of microbial communities as tools in forensic investigations such as determining the cause of death, estimating the time since death, or defining the microbiological profiles of individuals or samples that will be used to identify potential interpersonal or geographical links (Nodari et al. 2024). However, the presence and proliferation of microorganisms such as bacteria and fungi on cadavers can pose a risk, especially to individuals who come into direct contact with the remains. The possibility of transferring microorganisms such as bacteria, viruses, or prions from corpses to people who come into contact with them is well known (Geoffray et al. 2024). Additionally, several studies have highlighted the danger associated with the intensive growth of potentially pathogenic fungi on corpses (Schwarz et al. 2015; Szleszkowski et al. 2022; Martínez-Ramírez et al. 2013). These studies have isolated species classified as risk group 2, with allergenic potential that, in predisposed individuals, may also cause more severe infections. Microorganisms classified as Biosafety Level 2 (BSL2) pose a moderate risk to laboratory workers and the environment. They are usually native species associated with diseases of varying severity (CDC 2025, https://www.cdc.gov/training/quicklearns/biosafety).

Importantly, species with increasing drug resistance are frequently isolated from cadavers (Colombo et al. 2017; Schwarz et al. 2015; Tranchida et al. 2018). Drug resistance among microorganisms is primarily driven by the inappropriate and excessive use of drugs by humans (Revie et al. 2018; Ventola 2015). In addition, the use of fungicides in agriculture leads to the selection of environmentally resistant strains such as *Aspergillus fumigatus,* which can then infect humans (Burks et al. 2021). The widespread use of antibiotics in livestock contributes to the development of antibiotic resistance and may also have a negative impact on the environmental microbiome (Ventola 2015). The increase in drug resistance among microorganisms results in difficulties in treatment due to the ineffectiveness of standard therapy and the spread of resistant strains. An additional challenge is the limited development of novel antimicrobial agents (Revie et al. 2018; Ventola 2015). The limited development of new antimicrobial drugs is mainly due to financial issues, declining interest from pharmaceutical companies, and difficulties in obtaining effective drugs (Farha et al. 2025). The combination of limited antimicrobial drug development and the growing prevalence of antimicrobial resistance leads to concerning projections for the future, with potentially severe implications for public health and clinical practice (Antimicrobial Resistance Collaborators 2022). In forensic and environmental contexts, drug-resistant microorganisms pose an additional biosafety threat to personnel. However, to the best of our knowledge, the antimicrobial susceptibility profiles of microorganisms isolated from human corpses have not yet been systematically investigated.

The objective of this study was to characterize the micro- and mycobiome of human cadavers in an active decay stage of decomposition exhibiting visible fungal growth. The body had been exposed outdoors for an extended period, within a gazebo structure. Notably, extensive evidence of postmortem animal scavenging was observed. The presence of actively sporulating fungi combined with animal contact, may significantly increase the potential for microbial transmission. We hypothesized that outdoor-decomposed human cadavers may harbor previously unreported fungal species with potential pathogenicity and drug resistance. Therefore, to evaluate potential public health implications, antimicrobial susceptibility testing was performed on selected isolates using therapeutically relevant antimicrobial agents.

## Materials and methods

### Case report

On 30 March 2023, the body of a 56-year-old man was discovered lying on a bed in an open gazebo, with visible mold growth present. Prior to examination and autopsy, the body was stored under refrigerated conditions at 4 °C for 17 days in the Department of Forensic Medicine at Wroclaw Medical University.

### Sample collection

Samples were collected from the corpse during the autopsy on April 17, 2023, following standard microbiological procedures, i.e. using a viscose swab moistened with saline (0.85% NaCl) (Spychała et al. 2024). A total of 27 swabs were taken from 23 parts of the body exhibiting macroscopically visible mycelium, both from the surface of the corpse and from internal organs. The samples were stored at 5 ± 0.5 °C (for approximately 3 days) in transport tubes (Spychała et al. 2024), after which fungal isolation from the collected material was initiated.

### Isolation of microorganisms from samples

To isolate microorganisms from the corpse, the tips of the swabs containing the collected material were aseptically cut and placed into sterile conical polypropylene tubes (25 mL) with caps (FL Medical, Torreglia, Italy) containing 10 mL of physiological saline. The tubes were shaken at room temperature for approximately 5 min to facilitate the release of fungal and bacterial cells into the solution. Subsequently, 100 µL of the suspension was evenly distributed onto culture plates in triplicate to ensure uniform inoculation of the media.

### Isolation of fungi from samples

For fungal cultivation, four different culture media were utilized:

- Modified Leeming–Notman agar (MLNA) (consisting of: 1% peptone, 1% glucose, 0.2% yeast extract, 0.8% ox bile, 0.05% glycerol monostearate, 1% glycerol, 0.5% Tween 60, 2% olive oil and 1.5% agar) for the isolation of *Malassezia*;
- Potato Dextrose Agar (PDA) (Biomaxima, Lublin, Poland);
- Sabouraud’s Glucose Agar (SGA) (consisting of: 10 g·L^−1^ peptone, 40 g·L^−1^, glucose, 15 g·L^−1^ agar);
- Yeast Peptone Glucose (YPG) medium (consisting of: 20 g·L^−1^ peptone, 20 g·L^−1^ glucose, 20 g·L^−1^ agar, 10 g·L^−1^ yeast extract) reduced to 4.5 pH (YPG pH 4.5) and containing chloramphenicol (50 mg·L^−1^) to inhibit the growth of bacteria and some filamentous fungi according to Spychała et al. (2024).

The samples were incubated for 7 to 30 days at 5 ± 0.5 °C, 24 ± 0.5 °C and 37 ± 0.5 °C. The use of four different culture media and incubation at three distinct temperatures supported the isolation of the broadest possible spectrum of cultivable microscopic fungi from the corpse.

Subsequently, pure cultures were obtained by the single spore method on PDA medium employed for filamentous fungi and YPD medium for yeast-like fungi. Additionally, the isolates were preserved on PDA slants (filamentous fungi) and in 17% glycerol at −70 ± 0.1 °C (yeast-like fungi).

### Isolation of bacteria from samples

For bacteria cultivation, six different culture media were used, according to WHO (World Health Organization) (2003) protocols:

- Tryptone Soya Agar (TSA) (Biomerieux, Marcy-l'Étoile, France);
- Columbia Agar with 5% Sheep Blood (COS) (Biomerieux, Marcy-l'Étoile, France);
- MacConkey agar (MC) (Biomerieux, Marcy-l'Étoile, France);
- *Salmonella-Shigella* agar (SS) (Biomerieux, Marcy-l'Étoile, France);
- Chap—Mannitol Salt Agar (Chapman Medium) (Biomerieux, Marcy-l'Étoile, France);
- Schaedler broth (Biomerieux, Marcy-l'Étoile, France).

The cultures were incubated for 48 h at 28 ± 0.5 °C and for 18–24 h at 37 ± 0.5 °C. For the isolation of anaerobic bacteria, the samples were reductively plated on Shaedler Agar medium supplemented with 5% sheep blood and suspended in Shaedler Broth with vitamin K3. The plates were subsequently incubated under strict anaerobic conditions at 37 ± 0.5 °C for 48 h. In addition, the isolates were stored in 20% glycerol at −70 ± 0.1 °C.

### Fungal identification

The identification was performed using a combination of classical and molecular methods. Classical identification involved macro- and micromorphological observation**.** Additionally, molecular identification was performed. For this purpose DNA from fresh fungal cultures (maximum 2 week cultures) was extracted using the Bead-Beat Micro AX Gravity kit (A&A Biotechnology, Gdańsk, Poland) according to the manufacturer’s instructions. The polymerase chain reaction (PCR) was performed using one of two pairs of fungal-specific primers:

- ITS1 (5′-TCCGTAGGTGAACCTGCGG-3′) and ITS4 (5′-TCCTCCGCTTATTGATGC-3′) for amplifying the internal transcribed spacer (ITS) region of fungal DNA (White et al. 1990).
- NL-1 (5’-GCATATCAATAAGCGGAGGAAAAG-3’) and NL-4 (5’-GGTCCGTGTTTCAAGACGG-3’) for amplifying the D1/D2 region at the 5' end of the large subunit (LSU) rDNA gene (Kurtzman and Robnett 1997).

PCR was conducted using a T100 thermocycler (Bio-Rad, Berkeley, California, USA) according to protocols by White et al. (1990) and Kurtzman and Robnett (1997), respectively. The PCR product was purified using the Clean-Up Kit (A&ABiotechnology, Gdańsk, Poland) and subsequently sequenced at Macrogen Europe (Amsterdam, Netherlands, http://dna.macrogen.com/eng/).

### Bacteria identification

Bacterial isolates were identified based on biochemical characteristics and molecular methods. Classical identification of aerobic isolates was performed using chromogenic plates and manual tests. Gram-negative bacteria were identified using VITEK 2 Compact GN (Biomerieux, Marcy-l'Étoile, France) and Gram-positive bacteria using VITEK 2 Compact GP (Biomerieux, Marcy-l'Étoile, France) cards. In the case of anaerobic bacteria, after incubation, the isolates were plated on chromogenic agar and identified using the Rapid ANA II test (Thermo Fisher Scientific, Waltham, MA, USA).

Molecular identification commenced with the extraction of DNA from fresh bacterial cultures using the Bead-Beat Micro AX Gravity kit (A&A Biotechnology, Gdańsk, Poland) according to the manufacturer’s instructions. The polymerase chain reaction was performed using bacteria-specific primer 27 F (5’–AGAGTTTGATYMTGGCTCAG–3’) and 534R (5’–ATTACCGCGGCTGCTGG–3’) used to amplify the V1–V3 regions of the 16S rRNA gene (Abellan-Schneyder et al. 2021). PCR was performed using a T100 thermal cycler (Bio-Rad, Berkeley, CA, USA) following the protocol described by Abellan-Schneyder et al. (2021). The resulting PCR amplicons were purified with the Clean-Up Kit (A&A Biotechnology, Gdańsk, Poland) and subsequently sequenced at Macrogen Europe (Amsterdam, The Netherlands; http://dna.macrogen.com/eng/).

### Data analyses

Raw sequences were analyzed using the BioEdit Sequence Alignment Editor, and the resulting PCR products were compared with sequences available in GenBank of the National Center for Biotechnology Information (NCBI, Bethesda, Rockville, MD, USA) using the BLAST algorithm. The obtained sequences were subsequently submitted to the mentioned database. The isolates were deposited in the collection of the Department of Mycology and Genetics, University of Wrocław.

### Fungal drug sensitivity

Fifteen fungal isolates capable of growth at 37 °C (to reflect human body temperature) were selected for testing their sensitivity to antifungal drugs. Minimal inhibitory concentrations for voriconazole and amphotericin B (Sigma-Aldrich, St. Louis, USA) were tested according to EUCAST protocols for yeasts and molds (EUCAST 2024). Briefly, yeasts (*Candida albicans*, *Yarrowia deformans* UWR_721 and UWR_722, *Yarrowia lypolitica*, and *Debaryomyces hansenii*) suspensions were prepared from fresh culture on PDA plates in physiological salt solution to obtain an optical density (OD) of 0.5 McFarland. To prepare spore suspensions (*Penicillium polonicum*, *Penicillium expansum*,* Penicillium chrysogenum*,* Penicillium vinaceum*, *Penicillium crustosum*,* Aspergillus fumigatus*, *Scopulariopsis brevicaulis*, *Scopulariopsis crassa*, *Chaetomium subaffine*, and* Fusarium poae*) hyphae grown on PDA plates were suspended in 10 mL of physiological saline containing 0.1% Tween-20 using sterile swabs, transferred to a tube and left for 15 min. Then, OD was set at 0.5 McFarland. Both spore and yeast suspensions were diluted 1:10. Antibiotic solutions were prepared in 2xSD (synthetic defined; yeast nitrogen base 13.4 g L^−1^, glucose 40 g L^−1^) minimal medium as described in Rewak-Soroczyńska et al. (2021). Voriconazole and amphotericin B were dissolved in 2xSD to obtain a final test concentration range of 0.004–4 mg L^−1^. Fungal suspensions (0.1 mL) and antibiotic solutions (0.1 mL) were mixed in wells of a 96-well microtiter plate. Fungal suspension mixed with pure 2xSD constituted a growth control; antibiotic solutions mixed with pure physiological saline were blank controls. Each antibiotic concentration for each tested fungus as well as the growth controls was prepared in triplicates. Plates were incubated for 24–48 h at 37 ± 0.1 °C and optical density was measured at 600 nm using a BioTek® 800 absorbance reader TS (Agilent Technologies, Santa Clara, CA, USA). The experiment was repeated twice.

### Bacterial drug sensitivity

The antibiotic susceptibility of the tested bacterial strains was determined by the Kirby-Bauer diffusion-circulatory method, according to the recommendations of EUCAST (The European Committee on Antimicrobial Susceptibility Testing) (EUCAST 2025). For aerobic strains, a suspension of bacteria with a density of 0.5 on the McFarland scale (10⁸ cfu mL^−1^—colony forming unit—unit specifying the number of microorganisms in the test material) in sterile NaCl solution (0.9%) was applied with a sterile swab to the surface of MHA medium (BIOCORP, Paris, France). Then (after 15 min) discs with appropriate antibiotics (BBL Sensi-Disc, Becton Dickinson, Franklin Lakes, NJ, USA) were applied to the plate and incubated under oxygen, for 18 h at 37 ºC. For anaerobic bacterial strains (*Clostridioides difficile*) a prepared bacterial suspension with a density of 1.0 on the McFarland scale (10^5^ colony-forming unit mL^−1^) was applied with a sterile swab to the surface of Fastidious Anaerobe Agar + 5% defibrinated horse blood (FAA-HB), After 15 min discs with appropriate antibiotics (BBL Sensi-Disc, Becton Dickinson, Franklin Lakes, NJ, USA) were applied to the plate according to CLSI (CLSI 2025, Clinical and Laboratory Standards Institute, M100 Performance Standards for Antimicrobial Susceptibility Testing) recommendations for this group of bacteria. The medium with the applied discs was incubated in an anaerobic environment, at 35–37 ºC for 42–48 h.

Drug sensitivity was read by measuring the diameter (mm) of the growth inhibition zones of the bacterial culture around the discs. The size of the zone was compared with the interpretive standards for cutoff values for the strains tested given by EUCAST. Strains were divided into three categories: susceptible, intermediate susceptibility and resistant. Antibiotics used for drug susceptibility testing are listed in Table 1.

**Table 1:** Antibiotic discs used in the study, their abbreviations and concentrations

Abbreviation	Antibiotic	Concentration (μg/disk)
FOX	Cefoxitin	30
ERM	Erythromycin	15
CLD	Clindamycin	2
SXT	Trimethoprim-sulfamethoxazole	1.25 + 23.75
NOR	Norfloxacin	10
CIP	Ciprofloxacin	5
GEN	Gentamycin	10/30 for HLAR^1^ phenotype
AK	Amikacin	30
AMC	Amoxicillin-clavulanic acid	20 + 10
FEP	Cefepime	30
CAZ	Ceftazidime	10
CXM	Cefuroxime	30
TGC	Tigecycline	15
TZP	Piperacillin-tazobactam	30 + 6
IMP	Imipenem	10
AMP	Ampicillin	2
MTZ	Metronizadole	5
VA	Vanomycin	5

^1^HLAR – high-level aminoglycoside resistance

## Results

### Autopsy findings

The body exhibited signs of putrefactive decomposition as follows:

- deep reddish and greenish discoloration and softening of the skin layers;
- laminar detachment of flaccid gray epidermis;
- obliteration of the anatomical structure of internal organs with their softening (including the brain);
- reddish discoloration of the arterial walls with blood pigment (imbibition).

Additionally, there were extensive signs of postmortem animal scavenging (most likely rodents) in the form of soft tissue defects on the face and right upper limb of the deceased. Numerous, partially dried lice were also found on the corpse and clothing.

A large amount of white mycelium up to 0.5 cm thick and flat whitish-green mycelium was found on the skin of almost the entire body. Mold also partially covered the deceased's clothing. Thick deposits of fluffy gray-green fungal growth were also present in the entire respiratory tract from the larynx to the bronchi. No injuries resulting in death were revealed during the autopsy. The cause of death has not been determined. Advanced chronic lesions were found: cardiovascular system in the form of severe atherosclerosis of the aorta and coronary arteries, severe fatty liver, and fatty pancreas.

### Mycological analyses

The fungal isolates obtained during the analyses were divided into 103 groups exhibiting phenotypic differences (macro- and micromorphology) (Table 2), from which one representative was selected for genetic testing (isolates from UWR_610 to UWR_630, from UWR_632 to UWR_646, from UWR_648 to UWR_672, UWR_674, from UWR_676 to UWR_687, from UWR_689 to UWR_699 and from UWR_710 to UWR_727). Twenty-six different fungal species were identified, belonging to 10 families of both filamentous and yeast-like fungi (Table 2). Most isolates (99 isolates) belonged to the *Ascomycota* phylum, with only four isolates (from UWR_625 to UWR_628) belonging to the *Mucoromycota* phylum.

**Table 2:** Fungi isolated from cadaver and their pathogenicity to humans and instances (NA indicated no available information)

Fungi isolated from cadaver				Identity with sequence from GenBank^1^				Pathogenicity to humans
Isolate number	Identified species	Phylum	Family	GenBank accession No	The sequence length (bp)	Identity, %	Accession	
UWR_610	*Aspergillus fumigatus*	*Ascomycota*	*Aspergillaceae*	PV843833	505	100	OM259226.1	Opportunistic human pathogen (cutaneous, subcutaneous and invasive infections) (Liu et al. 2017; Tan et al. 2018; Rhodes et al. 2022)
UWR_611	*Botryotrichum domesticum*	*Ascomycota*	*Chaetomiaceae*	PV843834	492	100	MH899168.1	NA
UWR_612	*Candida albicans*	*Ascomycota*	*Debaryomycetaceae*	PV841877	514	100	MT193524.1	Opportunistic human pathogen (cutaneous and invasive infections) (Talapko et al. 2021)
UWR_613	*Chaetomium subaffine*	*Ascomycota*	*Chaetomiaceae*	PV843835	495	100	MN264617.1	NA
UWR_614	*Debaryomyces hansenii*	*Ascomycota*	*Debaryomycetaceae*	PV841878	516	100	KY511968.1	Opportunistic human pathogen (cutaneous, subcutaneous and invasive infections) (Desnos-Ollivier et al. 2008)
UWR_615				PV841879	511	100	KY511968.1	
UWR_616				PV841880	480	100	MT422082.1	
UWR_617				PV841881	407	100	KC111444.1	
UWR_618				PV841882	528	100	MT422082.1	
UWR_619				PV841883	545	100	KY512184.1	
UWR_620				PV841884	473	100	KC111444.1	
UWR_621				PV841885	519	100	MT180741.1	
UWR_622				PV841886	551	100	MT422082.1	
UWR_623				PV841887	547	100	MT422082.1	
UWR_624	*Fusarium poae*	*Ascomycota*	*Nectriaceae*	PV843836	369	100	MH299920.1	Mycotoxin producing species (Stenglein 2009)
UWR_625	*Helicostylum pulchrum*	*Mucoromycota*	*Thamnidiaceae*	PV843837	386	100	KC008791.1	NA
UWR_626	*Mortierella polycephala*	*Mucoromycota*	*Mortierellaceae*	PV843838	586	100	MH860490.1	NA
UWR_627				PV843839	573	100	MH860490.1	
UWR_628	*Mucor racemosus*	*Mucoromycota*	*Mucoraceae*	PV843840	400	100	PV156933.1	Opportunistic human pathogen (cutaneous and invasive infections) (Walther et al. 2019; Hai et al. 2024)
UWR_629	*Penicillium allii*	*Ascomycota*	*Aspergillaceae*	PV843841	502	100	MK450672.1	NA
UWR_630				PV843842	444	100	MK450672.1	
UWR_632	*Penicillium chrysogenum*	*Ascomycota*	*Aspergillaceae*	PV843843	516	100	MG022175.1	Opportunistic human pathogen (cutaneous, subcutaneous and invasive infections). Allergen-inducing species (Geltner et al. 2013). Mycotoxin producing species (García-Estrada et al. 2011)
UWR_633				PV843844	459	100	MT328526.1	
UWR_634				PV843845	416	100	MT328526.1	
UWR_635				PV843846	392	100	MT328526.1	
UWR_636				PV843847	362	100	MK460375.1	
UWR_637				PV843848	505	100	PP053032.1	
UWR_638				PV843849	498	100	KX263830.1	
UWR_639				PV843850	510	100	PP053032.1	
UWR_640				PV843851	452	100	PP053032.1	
UWR_641	*Penicillium commune*	*Ascomycota*	*Aspergillaceae*	PV843852	502	100	MK660351.1	NA
UWR_642				PV843853	523	100	KY646438.1	
UWR_643				PV843854	517	100	KU936231.1	
UWR_644				PV843855	487	100	MT558930.1	
UWR_645				PV843856	523	100	KY646438.1	
UWR_646				PV843857	487	100	MT558930.1	
UWR_648				PV843858	391	100	KC009817.1	
UWR_649				PV843859	492	100	MT558930.1	
UWR_650				PV843860	509	100	MK660351.1	
UWR_651				PV843861	357	100	KC009817.1	
UWR_652				PV843862	489	100	MT558930.1	
UWR_653				PV843863	422	100	KM115154.1	
UWR_654				PV843864	485	100	MT558930.1	
UWR_655				PV843865	490	100	MT558930.1	
UWR_656				PV843866	475	100	MT558930.1	
UWR_657				PV843867	447	100	MT558930.1	
UWR_658				PV843868	489	100	MT558930.1	
UWR_659				PV843869	486	100	MT558930.1	
UWR_660				PV843870	513	100	MT558930.1	
UWR_661				PV843871	519	100	MK660352.1	
UWR_662				PV843872	508	100	MT558930.1	
UWR_663				PV843873	485	100	KC009817.1	
UWR_664				PV843874	487	100	MT558930.1	
UWR_665				PV843875	379	100	KC009817.1	
UWR_666	*Penicillium crustosum*	*Ascomycota*	*Aspergillaceae*	PV843876	414	100	KC354488.1	Mycotoxin producing species (Prencipe et al. 2018)
UWR_667				PV843877	447	100	MF072618.1	
UWR_668	*Penicillium discolor*	*Ascomycota*	*Aspergillaceae*	PV843878	490	100	LR758010.1	NA
UWR_669				PV843879	523	100	MT832027.1	
UWR_670	*Penicillium expansum*	*Ascomycota*	*Aspergillaceae*	PV843880	490	100	MT582774.1	Mycotoxin producing species (Sanzani et al. 2012)
UWR_671	*Penicillium fuscoglaucum*	*Ascomycota*	*Aspergillaceae*	PV843881	500	100	MT558936.1	NA
UWR_672	*Penicillium magnielliptisporum*	*Ascomycota*	*Aspergillaceae*	PV843882	492	100	OP179044.1	NA
UWR_674	*Penicillium polonicum*	*Ascomycota*	*Aspergillaceae*	PV843883	492	100	MT582786.1	Mycotoxin producing species (Frisvad et al. 2004)
UWR_676				PV843884	531	100	KF938446.1	
UWR_677				PV843885	352	100	MZ901197.1	
UWR_678				PV843886	504	100	MN589643.1	
UWR_679				PV843887	521	100	MT582786.1	
UWR_680				PV843888	405	100	MN589643.1	
UWR_681				PV843889	357	100	MZ901197.1	
UWR_682				PV843890	508	100	MT529189.1	
UWR_683				PV843891	498	100	OQ852949.1	
UWR_684				PV843892	510	100	MT582786.1	
UWR_685				PV843893	356	100	MZ901197.1	
UWR_686				PV843894	479	100	MT582786.1	
UWR_687	*Penicillium solitum*	*Ascomycota*	*Aspergillaceae*	PV843895	495	100	MH860761.1	NA
UWR_689				PV843896	478	100	EU645698.1	
UWR_690				PV843897	361	100	ON751772.1	
UWR_691				PV843898	494	100	MT913575.1	
UWR_692				PV843899	472	100	MK761050.1	
UWR_693				PV843900	365	100	LR758011.1	
UWR_694	*Penicillium vinaceum*	*Ascomycota*	*Aspergillaceae*	PV843901	466	100	MT312765.1	NA
UWR_695	*Pseudogymnoascus pannorum*	*Ascomycota*	*Pseudeurotiaceae*	PV843902	450	100	MH859889.1	NA
UWR_696				PV843903	491	100	LT549077.1	
UWR_697				PV843904	436	100	MH864756.1	
UWR_698				PV843905	412	100	MH864091.1	
UWR_699				PV843906	456	100	MH864091.1	
UWR_710				PV843907	463	100	MH469520.1	
UWR_711				PV843908	452	100	MH859889.1	
UWR_712				PV843909	479	100	OR900531.1	
UWR_713				PV843910	485	100	MH864091.1	
UWR_714				PV843911	391	100	MH854616.1	
UWR_715	*Scopulariopsis brevicaulis*	*Ascomycota*	*Microascaceae*	PV843912	500	100	OR760546.1	Opportunistic human pathogen (cutaneous, subcutaneous and invasive infections) (Steinbach et al. 2004)
UWR_716				PV843913	389	100	OR760546.1	
UWR_717				PV841888	529	100	KJ443117.1	
UWR_718	*Scopulariopsis crassa*	*Ascomycota*	*Microascaceae*	PV841889	369	100	KU746750.1	NA
UWR_719	*Scopulariopsis fusca*	*Ascomycota*	*Microascaceae*	PV841890	520	100	LN850836.1	Opportunistic human pathogen (cutaneous infections) (Issakainen et al. 2007)
UWR_720	*Yarrowia deformans*	*Ascomycota*	*Dipodascaceae*	PV841891	410	100	MK394171.1	NA
UWR_721				PV841892	402	100	MK394171.1	
UWR_722				PV841893	472	100	MK394171.1	
UWR_723	*Yarrowia lipolytica*	*Ascomycota*	*Dipodascaceae*	PV841894	474	100	MT151658.1	Opportunistic human pathogen (cutaneous, subcutaneous and invasive infections) (Zieniuk and Fabiszewska 2018)
UWR_724				PV841895	487	100	MH545931.1	
UWR_725				PV841896	437	100	MT151658.1	
UWR_726				PV841897	431	100	MT151658.1	
UWR_727				PV841898	484	100	MT151658.1	

^1^All E values were zero and all Query Cover values were 100%. Sequences were obtained using the primer pair ITS1 and ITS4, except for isolates UWR_612, from UWR_614 to UWR_623 and from UWR_717 to UWR_727 for which the primer pair NL-1 and NL-4 was used

Based on BLAST analysis, all sequences had an E-value of zero and 100% query coverage and percent identity. The sequence lengths ranged from 352 to 586 bp. The fungal rDNA nucleotide sequences obtained in the study were submitted to GenBank under accession numbers from PV843833 to PV843913 (sequences amplifying the internal transcribed spacer (ITS) region) and from PV841877 to PV841898 (sequences amplifying the D1/D2 region at the 5' end of the large subunit (LSU) rDNA gene).

In this case, we focused on comparing the necromicrobiome (microorganisms isolated from outside the body) and the thanatomicrobiome (microorganisms isolated from inside the body). Therefore, the sampling sites were divided into areas corresponding to each group. The necromicrobiome (face, left temporal region, mental region, right auricle, right hand, left hand, chest, abdominal region, back, thigh and right knee, thigh and left knee, right calf first colony type, right calf second colony type, right calf third colony type, left calf, feet) and the thanatomicrobiome (trachea, lungs, diaphragmatic pleura, liver, liver parenchyma, porta hepatis, intestinal vasculature) were presented in Table 3. For each sampling location, fungal growth was assessed on four different culture media. Species occurrence was recorded as presence or absence on each medium, and the total number of positive isolations was used to evaluate their distribution and frequency.

**Table 3:** Fungi isolates cultured from cadaver based on material collection site and isolation conditions, i.e. a specific temperature and culture medium

Isolate number	Species^1^	5 °C				25 °C	
		YPG ^pH 4.5^	PDA	SGA	MLNA	YPG ^pH 4.5^	PDA
UWR_610	*Aspergillus fumigatus*					+ ^15^	
UWR_611	*Botryotrichum domesticum*					+ ^6, 16, 17, 20, 22^	+ ^6^
UWR_612	*Candida albicans*						
UWR_613	*Chaetomium subaffine*						+ ^19^
UWR_614	*Debaryomyces hansenii*						+ ^11^
UWR_615		+ ^8, 12^		+ ^10, 19^		+ ^8, 12^	+ ^12^
UWR_616			+ ^23^	+ ^19^			+ ^19^
UWR_617		+ ^12^	+ ^10, 16^				
UWR_618		+ ^12^	+ ^12^				
UWR_619		+ ^8^	+ ^10, 11^			+ ^3, 10, 21, 13, 15, 16, 21^	+ ^10, 13, 15, 20^
UWR_620		+ ^10^		+ ^10^			
UWR_621		+ ^12^					
UWR_622		+ ^4, 9, 11, 12, 23^	+ ^23^	+ ^23^	+ ^23^	+ ^1, 2, 5, 7, 10, 12, 19, 21, 23^	+ ^1, 2, 6, 7, 12, 22^
UWR_623		+ ^3, 4, 10, 13^	+ ^13^	+ ^13^	+ ^13^	+ ^3, 6, 9, 11, 13, 14, 15, 17^	+ ^6, 9, 10, 13, 15, 23^
UWR_624	*Fusarium poae*					+ ^2^	+ ^2^
UWR_625	*Helicostylum pulchrum*	+ ^2, 3, 6, 7, 9, 14, 15, 16, 19, 21, 22, 23^	+ ^2, 4, 6, 7, 9, 12, 15, 16, 18, 19, 21, 22^	+ ^2, 4, 6, 7, 9, 12, 15, 16, 17, 18, 19, 21, 22^	+ ^2, 6, 7, 12, 14, 15, 19, 20, 21, 22^	+ ^7^	+ ^7^
UWR_626	*Mortierella polycephala*					+ ^15^	
UWR_627		+ ^3, 5, 15, 20^		+ ^15, 16, 20^	+ ^15^	+ ^2, 3, 5, 15, 18, 19, 20, 21^	+ ^2, 5, 18^
UWR_628	*Mucor racemosus*	+ ^7^	+ ^7^	+ ^7^	+ ^7^		+ ^13, 19, 20^
UWR_629	*Penicillium allii*						
UWR_630		+ ^4^	+ ^23^	+ ^23^	+ ^23^		
UWR_632	*Penicillium chrysogenum*						
UWR_633						+ ^5^	+ ^5^
UWR_634							
UWR_635							
UWR_636							
UWR_637							
UWR_638							
UWR_639							
UWR_640							
UWR_641	*Penicillium commune*	+ ^7, 8, 18^	+ ^7, 18^	+ ^7, 18^	+ ^7, 14, 18^		
UWR_642						+ ^12^	
UWR_643						+ ^14^	
UWR_644						+ ^3^	+ ^3, 5, 12^
UWR_645		+ ^4^		+ ^12^	+ ^4^		
UWR_646							+ ^1^
UWR_648		+ ^21^		+ ^3, 14, 21^			
UWR_649		+ ^4, 6, 18, 21, 22^	+ ^1, 6, 12, 15, 18, 22^	+ ^1, 6, 15, 17, 18, 21, 22^	+ ^1, 4, 5, 15, 18, 22^		
UWR_650							
UWR_651			+ ^8, 14, 20^		+ ^14^	+ ^5^	+ ^5, 16^
UWR_652			+ ^3^		+ ^3^		
UWR_653		+ ^7^	+ ^7^	+ ^7^	+ ^6, 7^	+ ^18^	+ ^1, 16, 18^
UWR_654		+ ^4, 22^	+ ^22^	+ ^22^	+ ^22^		
UWR_655		+ ^22^	+ ^22^	+ ^22^	+ ^22^	+ ^22^	
UWR_656		+ ^19^	+ ^19^	+ ^19^	+ ^19^		
UWR_657						+ ^7, 17^	+ ^7, 17^
UWR_658						+ ^22^	
UWR_659						+ ^2^	+ ^2^
UWR_660						+ ^5^	+ ^5, 19, 20^
UWR_661			+ ^20^				
UWR_662						+ ^15, 23^	+ ^1, 5, 15, 19, 20^
UWR_663						+ ^22^	+ ^19, 22^
UWR_664		+ ^6, 12, 19^	+ ^6, 15, 19, 20^	+ ^5, 6, 14, 17, 19, 20^	+ ^19^		+ ^22^
UWR_665		+ ^8, 21^	+ ^2^	+ ^17, 21, 23^	+ ^23^		+ ^12^
UWR_666	*Penicillium crustosum*				+ ^14^	+ ^14^	+ ^14^
UWR_667							
UWR_668	*Penicillium discolor*					+ ^2^	+ ^2^
UWR_669		+ ^19^	+ ^8, 19^	+ ^7, 19^	+ ^5, 13, 19^		+ ^2^
UWR_670	*Penicillium expansum*						+ ^6^
UWR_671	*Penicillium fuscoglaucum*			^+20^	+ ^20^		
UWR_672	*Penicillium magnielliptisporum*		+ ^16^				+ ^16^
UWR_674	*Penicillium polonicum*	+ ^4^	+ ^12, 14^	+ ^11^	+ ^4, 9, 11, 14^	+ ^5^	+ ^5^
UWR_676		+ ^23^	+ ^23^	+ ^23^	+ ^23^		
UWR_677			+ ^9^	+ ^9^		+ ^14^	+ ^14^
UWR_678		+ ^8, 11, 17, 20^	+ ^1, 2, 5, 8, 14, 15, 17^	+ ^1, 3, 5, 12, 14, 15, 17, 23^	+ ^1, 4, 12, 14, 15, 17, 23^	+ ^2, 9, 11, 12, 16, 20^	+ ^2, 9, 11, 12, 20, 21^
UWR_679		+ ^8, 21^	+ ^8, 12^	+ ^21^	+ ^8^	+ ^8^	+ ^8^
UWR_680			+ ^3, 15^		+ ^3^	+ ^4, 9, 13^	+ ^4, 6, 15, 16^
UWR_681			+ ^1^	+ ^1^	+ ^1^	+ ^6, 9, 14, 15^	+ ^6, 14, 15, 22^
UWR_682		+ ^4^			+ ^13^		
UWR_683							
UWR_684			+ ^5, 21^	+ ^5, 11^	+ ^11^	+ ^1, 8, 17, 18^	+ ^1, 8, 13, 17, 18^
UWR_685		+ ^4^	+ ^4, 13, 14^	+ ^4, 5, 13, 14^	+ ^4, 13^	+ ^1, 3, 8, 12, 13^	+ ^1, 3, 8, 13^
UWR_686		+ ^9, 11, 12^	+ ^3, 11, 17, 21^		+ ^3, 8, 12^	+ ^3, 4, 12, 13, 14, 15, 23^	+ ^3, 4, 6, 14, 21, 23^
UWR_687	*Penicillium solitum*	+ ^12^					
UWR_689							+ ^6, 15, 17, 19^
UWR_690						+ ^7^	+ ^7^
UWR_691							+ ^23^
UWR_692			+ ^16^				
UWR_693			+ ^2^			+ ^6, 7, 22^	+ ^2, 7, 22^
UWR_694	*Penicillium vinaceum*						+ ^17^
UWR_695	*Pseudogymnoascus pannorum*						+ ^16^
UWR_696							+ ^16^
UWR_697							+ ^16^
UWR_698						+ ^2, 20^	
UWR_699							+ ^2^
UWR_710		+ ^14^					
UWR_711		+ ^16^		+ ^16^	+ ^16^		+ ^17^
UWR_712				+ ^16^			
UWR_713				+ ^20^			
UWR_714						+ ^15^	+ ^15^
UWR_715	*Scopulariopsis brevicaulis*						
UWR_716						+ ^19^	
UWR_717						+ ^20^	
UWR_718	*Scopulariopsis crassa*	+ ^18, 23^	+ ^18^	+ ^18^	+ ^18^	+ ^1, 6, 13, 15, 16, 23^	+ ^16^
UWR_719	*Scopulariopsis fusca*						
UWR_720	*Yarrowia deformans*	+ ^7^	+ ^7^	+ ^7^	+ ^7^		
UWR_721							
UWR_722							
UWR_723	*Yarrowia lipolytica*						
UWR_724							
UWR_725			+ ^15^	+ ^15^	+ ^15^		
UWR_726							
UWR_727						+ ^6, 18^	+ ^18^

Isolate number	Species^1^	25 °C		37 °C			
		SGA	MLNA	YPG ^pH 4.5^	PDA	SGA	MLNA
			+ ^11^	+ ^15^	+ ^14, 17^	+ ^14, 17^	+ ^15, 17^
UWR_610	*Aspergillus fumigatus*	+ ^6^	+ ^16^				
UWR_611	*Botryotrichum domesticum*				+ ^16^		
UWR_612	*Candida albicans*			+ ^6, 15, 19, 21^	+ ^19, 21^		
UWR_613	*Chaetomium subaffine*	+ ^11^		+ ^12^			
UWR_614	*Debaryomyces hansenii*		+ ^12, 15^	+ ^12, 20^	+ ^10, 12, 15, 16^	+ ^15^	+ ^11, 15, 20^
UWR_615							
UWR_616							
UWR_617							
UWR_618		+ ^13, 16^	+ ^10, 16^	+ ^3, 10, 15, 16, 20^	+ ^3, 15, 16, 20^	+ ^3, 15, 16^	+ ^3, 10, 16, 20^
UWR_619					+ ^10^		
UWR_620							
UWR_621		+ ^19, 21^	+ ^2, 7, 19, 22^	+ ^2, 5, 7, 8, 11, 12, 19, 21, 22^	+ ^2, 5, 7, 8, 10, 12, 19, 20, 21, 22, 23^	+ ^2, 5, 7, 8, 12, 19, 20, 21, 22^	+ ^2, 5, 7, 8, 12, 21, 22^
UWR_622		+ ^9, 13, 23^	+ ^23^	+ ^4, 6, 8, 9, 10, 11, 13, 14, 15, 17, 23^	+ ^6, 8, 9, 10, 11, 13, 14, 15, 17, 23^	+ ^6, 8, 9, 11, 14, 15, 17, 23^	+ ^4, 9, 10, 11, 13, 15, 17, 23^
UWR_623				+ ^16^			
UWR_624	*Fusarium poae*	+ ^7, 21^	+ ^7^				
UWR_625	*Helicostylum pulchrum*						
UWR_626	*Mortierella polycephala*	+ ^5, 18^	+ ^5, 18^				
UWR_627		+ ^13^					
UWR_628	*Mucor racemosus*	+ ^7^					
UWR_629	*Penicillium allii*						
UWR_630				+ ^14^	+ ^14^	+ ^14^	
UWR_632	*Penicillium chrysogenum*	+ ^5^	+ ^5^	+ ^5^	+ ^5^	+ ^5^	+ ^5^
UWR_633					+ ^6^	+ ^6^	
UWR_634				+ ^20^			
UWR_635			+ ^19^				
UWR_636				+ ^3^	+ ^3, 6, 17, 22^	+ ^3, 6^	+ ^3^
UWR_637				+ ^5, 22^	+ ^5, 17, 22^	+ ^5, 19, 22^	+ ^1, 5, 19, 22^
UWR_638					+ ^15, 23^		
UWR_639				+ ^14^	+ ^23^		
UWR_640							
UWR_641	*Penicillium commune*						
UWR_642							
UWR_643		+ ^3^	+ ^3^				
UWR_644							
UWR_645							
UWR_646		+ ^7^					
UWR_648							
UWR_649		+ ^11^					
UWR_650		+ ^5^	+ ^5^				
UWR_651		+ ^19^					
UWR_652		+ ^18, 19, 23^	+ ^18^				
UWR_653							
UWR_654		+ ^7^					
UWR_655							
UWR_656		+ ^1, 7, 17^	+ ^7, 14, 17^				
UWR_657							
UWR_658							
UWR_659		+ ^5^	+ ^5^				
UWR_660							
UWR_661		+ ^5, 15 17, 19, 20^	+ ^15, 20^				
UWR_662		+ ^22^	+ ^22^				
UWR_663		+ ^22^	+ ^22^				
UWR_664		+ ^12^	+ ^12^				
UWR_665		+ ^14, 19^	+ ^14^	+ ^4, 14, 20^	+ ^4, 14, 17^	+ ^7, 14, 17^	+ ^4, 17^
UWR_666	*Penicillium crustosum*			+ ^14^	+ ^14^	+ ^14^	+ ^14, 19^
UWR_667		+ ^7^					
UWR_668	*Penicillium discolor*	+ ^16^					
UWR_669		+ ^6^			+ ^6^		
UWR_670	*Penicillium expansum*						
UWR_671	*Penicillium fuscoglaucum*						
UWR_672	*Penicillium magnielliptisporum*	+ ^5^	+ ^5^				
UWR_674	*Penicillium polonicum*	+ ^23^		+ ^11^			+ ^23^
UWR_676		+ ^14^	+ ^14^	+ ^11^		+ ^2^	+ ^14^
UWR_677		+ ^11, 12, 21^	+ ^9, 11, 12, 21^	+ ^4, 12^	+ ^2, 4, 8, 11, 12, 20, 21^	+ ^2, 4, 6, 8, 12, 21^	+ ^12^
UWR_678		+ ^8, 21^	+ ^8^	+ ^8, 12^			+ ^8^
UWR_679		+ ^4, 6, 9, 11, 15^	+ ^4, 6, 9, 13, 15^	+ ^4, 9, 13^	+ ^4, 9, 15^	+ ^4, 9, 11, 15^	+ ^9, 11, 15^
UWR_680		+ ^1, 6, 14, 15, 22^	+ ^6, 14, 15, 22^	+ ^7, 14, 18^	+ ^7, 14, 18, 22^	+ ^14, 18, 22^	+ ^14, 18, 22^
UWR_681							
UWR_682		+ ^23^					
UWR_683		+ ^8, 16, 17, 18, 21^	+ ^1, 8, 17, 18^	+ ^1, 11, 15, 18^	+ ^1, 8, 12, 15, 17^	+ ^8, 15, 17^	+ ^8, 17^
UWR_684		+ ^1, 3, 8, 13^	+ ^1, 3, 8, 13^	+ ^4, 13^	+ ^13^	+ ^13^	+ ^4, 13^
UWR_685		+ ^1, 3, 4, 6, 12, 14, 20^	+ ^3, 4, 6, 16, 23^	+ ^3, 12, 14, 15, 18, 23^	+ ^2, 3, 14, 15, 17, 18, 19, 23^	+ ^3, 11, 14, 18, 23^	+ ^3, 4, 6, 14, 15, 18, 21, 23^
UWR_686							
UWR_687	*Penicillium solitum*	+ ^1, 7, 17^	+ ^7, 17^				
UWR_689							
UWR_690		+ ^19^					
UWR_691							
UWR_692		+ ^19, 21^	+ ^9, 19^				
UWR_693							
UWR_694	*Penicillium vinaceum*						
UWR_695	*Pseudogymnoascus pannorum*						
UWR_696							
UWR_697							
UWR_698							
UWR_699							
UWR_710							
UWR_711							
UWR_712							
UWR_713							
UWR_714				+ ^19^			
UWR_715	*Scopulariopsis brevicaulis*			+ ^17, 19^			
UWR_716				+ ^20^			
UWR_717			+ ^23^	+ ^5, 16, 18, 23^	+ ^5, 15, 16^	+ ^5, 15^	+ ^5, 15^
UWR_718	*Scopulariopsis crassa*			+ ^14^	+ ^14^	+ ^14^	
UWR_719	*Scopulariopsis fusca*						
UWR_720	*Yarrowia deformans*			+ ^2, 20^			
UWR_721				+ ^12^			
UWR_722				+ ^17^			
UWR_723	*Yarrowia lipolytica*			+ ^19^			
UWR_724			+ ^19^	+ ^19^		+ ^19^	
UWR_725				+ ^4, 5, 17^	+ ^5, 17^	+ ^5, 7, 17^	+ ^5, 17^
UWR_726				+ ^1, 14, 18, 21^	+ ^18, 19, 21, 22^	+ ^18, 19, 21, 22^	+ ^6, 18, 23^

^1^A (+) sign indicates that the fungi have been isolated from a specific site. Labelling of individual material collection sites: 1. face, 2. left temporal region, 3. mental region, 4. trachea, 5. right auricle, 6. right hand, 7. left hand, 8. lungs, 9. diaphragmatic pleura, 10. liver, 11. liver parenchyma, 12. porta hepatis, 13. intestinal vasculature, 14. chest, 15. abdominal region, 16. back, 17. thigh and right knee, 18. thigh and left knee, 19. right calf first colony type, 20. right calf second colony type, 21. right calf third colony type, 22. left calf, 23. feet

**Fig. 1:**
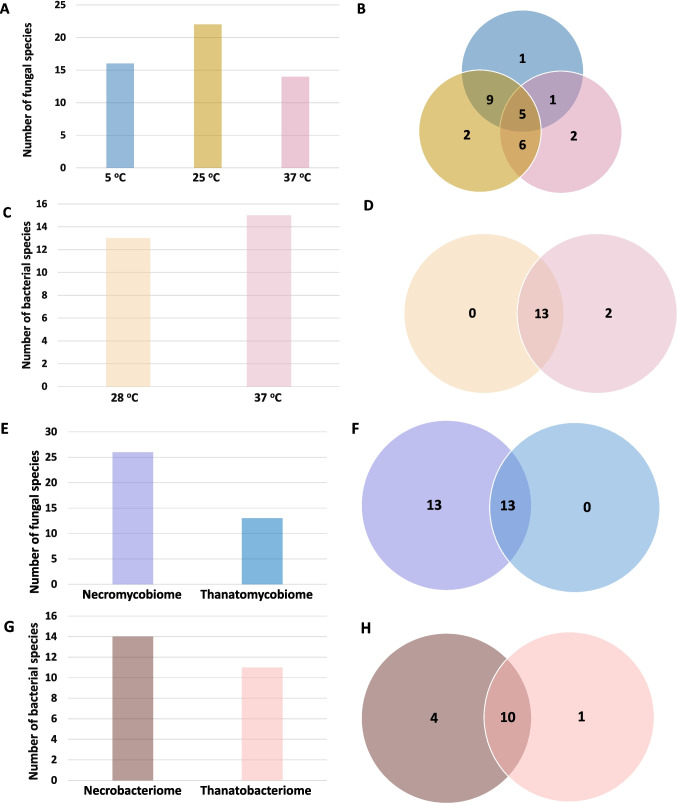
The number of fungal (**A**, **B**) and bacterial species (**C**, **D**) cultured depending on the incubation temperature and the number of fungal (**E**, **F**) and bacterial species (**G**, **H**) isolated from outside (necromycobiome/necrobacteriome) and inside (thanatomycobiome/thanatobacteriome) the body

Table 3 provides a detailed list of the isolates, categorized by sampling location and culture conditions. The most species-rich areas for fungi were the back, thigh, and right knee, as well as the first and second types of colonies on the right calf (13 species each). In general, anatomical sites from which isolates belong to the thanatomycobiome are colonized by a smaller number of fungal species (from 1 species: *D. hansenii* in the case of the liver to 7 in the case of the trachea). The largest number of species (22 species) was recovered at 25 °C, followed by 16 species at 5 °C and 14 species at 37 °C. Interestingly, five species were common to all three temperature conditions (Fig. 1A, B).

**Fig. 2:**
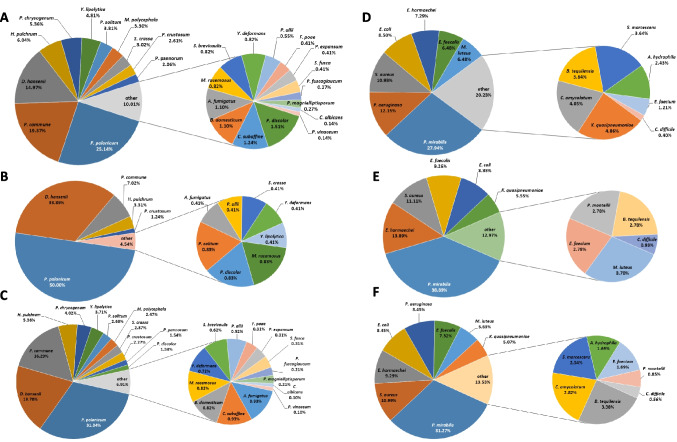
Percentage distribution of all fungal species belonging to the necromycobiome (**A**), thanatomycobiome (**B**), and total (**C**) and all bacterial species belonging to the necrobacteriome (**D**), thanatobacteriome (**E**), and total (**F**)

A total of 26 fungal species were identified both outside and inside the body of the deceased. The necromycobiome contained all 26 species, while the thanatomycobiome consisted of 13 species, also isolated from outside the body (Fig. 1E, F). The dominant species were *P. polonicum* (31.34%), *D. hansenii* (19.7%), and *Penicillium commune* (16.29%) (Fig. 2C). In the case of the necromycobiome (Fig. 2A) and the thanatomycobiome (Fig. 2B) the distribution of the three dominant species was similar.

There were isolates recorded only once, such as *C. albicans* (isolated from the back) and *P.* *vinaceum* (isolated from the right thigh and knee), accounting for 0.1% of all isolated fungi.

### Bacteriological analyses

Bacterial isolates obtained during the study were divided into 16 groups based on morphology. Representatives were selected for molecular testing, which allowed for the identification of 15 species (isolates from UWR_728 to UWR_743) belonging to 3 phyla (*Proteobacteria*, *Firmicutes*, and *Actinobacteria*) and 11 families.

Based on BLAST analysis, all sequences had an E-value of zero, and 100% query cover. The sequence lengths ranged from 352 to 462 bp and an identity range of 99.18–100%. The bacterial 16S rRNA nucleotide sequences were submitted to GenBank under accession numbers from PV843919 to PV843934 (Table 4).

**Table 4:** Bacteria isolated from cadaver and their pathogenicity to humans and instances (NA indicated no available information)

Bacteria isolated from cadaver				Identity with sequence from GenBank^1^				Pathogenicity to humans
Isolate number	Identified species	Phylum	Family	GenBank accession No	The sequence length (bp)	Identity, %	Accession	
UWR_728	*Aeromonas hydrophila*	*Proteobacteria*	*Aeromonadaceae*	PV843919	432	100	MG428794.1	Opportunistic human pathogen (cutaneous, subcutaneous and systemic infections) (Zhiyong et al. 2002)
UWR_729	*Bacillus tequilensis*	*Firmicutes*	*Bacillaceae*	PV843920	442	100	PQ162523.1	NA
UWR_730	*Clostridioides difficile*	*Firmicutes*	*Peptostreptococcaceae*	PV843921	396	100	CP118685.1	Opportunistic human pathogen (ranging from mild diarrhea to severe pseudomembranous colitis, and in extreme cases sepsa) (Fachi et al. 2024)
UWR_731	*Corynebacterium amycolatum*	*Actinobacteria*	*Corynebacteriaceae*	PV843922	353	100	JN571071.1	Opportunistic human pathogen (cutaneous and systemic infections) (Esteban et al. 1999; Oteo et al. 2001)
UWR_732	*Enterobacter hormaechei*	*Proteobacteria*	*Enterobacteriaceae*	PV843923	375	99.47	OQ421723.1	Opportunistic human pathogen (nosocomial, systemic infections (St. John et al. 2023)
UWR_733	*Enterococcus faecium*	*Firmicutes*	*Enterococcaceae*	PV843924	417	100	CP112658.1	Opportunistic human pathogen (nosocomial, systemic infections (Vu and Carvalho 2011)
UWR_734	*Enterococcus faecalis*	*Firmicutes*	*Enterococcaceae*	PV843925	462	99.78	OR185453.1	Opportunistic human pathogen (nosocomial, systemic infections (Vu and Carvalho 2011)
UWR_735	*Escherichia coli*	*Proteobacteria*	*Enterobacteriaceae*	PV843926	358	99.44	OL597932.1	Opportunistic human pathogen (systemic infections and food poisoning) (Nataro and Kaper 1998; Kim et al. 2002)
UWR_736	*Klebsiella quasipneumoniae*	*Proteobacteria*	*Enterobacteriaceae*	PV843927	439	99.77	LR134196.1	Opportunistic human pathogen (systemic infections) (Chew et al. 2021)
UWR_737	*Micrococcus luteus*	*Actinobacteria*	*Micrococcaceae*	PV843928	417	100	MZ338821.1	Opportunistic human pathogen (systemic infections) (Shi et al. 2023)
UWR_738	*Proteus mirabilis*	*Proteobacteria*	*Morganellaceae*	PV843929	357	99.72	OR838320.1	Opportunistic human pathogen (systemic infections, mainly urinary tract infections) (Armbruster et al. 2018)
UWR_739	*Pseudomonas aeruginosa*	*Proteobacteria*	*Pseudomonadaceae*	PV843930	363	99.18	OR574360.1	Opportunistic human pathogen (cutaneous, subcutaneous, systemic, invasive infections—common in hospitalized patients) (de Bentzmann and Plésiat 2011; Spernovasilis et al. 2021)
UWR_740	*Pseudomonas monteilii*	*Proteobacteria*	*Pseudomonadaceae*	PV843931	383	99.48	OL839966.1	Opportunistic human pathogen (systemic infections, mainly in hospitalized patients) (Lee et al. 2024)
UWR_741	*Serratia marcescens*	*Proteobacteria*	*Yersiniaceae*	PV843932	352	100	MK719775.1	Opportunistic human pathogen (cutaneous, subcutaneous, systemic, infections (Zivkovic et al. 2023)
UWR_742	*Staphylococcus aureus*	*Firmicutes*	*Staphylococcaceae*	PV843933	386	100	CP030498.1	Opportunistic human pathogen (cutaneous, subcutaneous, systemic, infections (Pollitt et al. 2018)
UWR_743				PV843934	442	100	LC090540.1	

^1^All E values were zero and all Query Cover values were 100%

Bacteria isolation was carried out at two temperatures. All 15 species were isolated at 37 °C, while 13 species were isolated at 28 °C. Interestingly, *C. difficile* and *Pseudomonas monteilii* were isolated only at 37 °C (Fig. 1C, D).

Table 5 presents the bacterial isolates in relation to culture conditions and sample location. Fourteen out of fifteen bacterial species were recorded from samples taken from outside the body. The only species found exclusively inside the body was *P. monteilii*, isolated from the trachea. The number of isolated species ranged from one (in the sample from the back) to eight (in the first colony type from the right calf). In contrast, eleven species were isolated from internal body samples, with the number of species ranging from three to six depending on the specific body region (Fig. 1G, H).

**Table 5:** Bacteria isolates cultured from cadaver based on material collection site and isolation conditions, i.e. a specific temperature and culture medium

Isolate number	Species^1^	28 °C					37 °C				
		TSA	COS	MC	Chap	SS	TSA	COS	MC	Chap	SS
UWR_728	*Aeromonas hydrophila*		+ ^3, 7^	+ ^3, 7^				+ ^3, 7^			
UWR_729	*Bacillus tequilensis*	+ ^4, 19^	+ ^2, 19^				+ ^2, 4, 6, 20^	+ ^2, 4, 6, 20^			
UWR_730	*Clostridioides difficile*							+ ^9, 15^			
UWR_731	*Corynebacterium amycolatum*	+ ^20, 21, 23^	+ ^20, 21, 23^				+ ^14, 23^	+ ^14, 23^			
UWR_732	*Enterobacter hormaechei*	+ ^3, 4, 11, 15^	+ ^3, 4, 11, 15^	+ ^3, 4, 11, 15^			+ ^1, 2, 3, 4, 7, 10, 13, 19^	+ ^1, 2, 3, 4, 7, 10, 13, 19^	+ ^1, 2, 3, 4, 7, 10, 13, 19^		
UWR_733	*Enterococcus faecium*		+ ^14, 10^				+ ^10, 14^	+ ^10, 14^			
UWR_734	*Enterococcus faecalis*	+ ^1, 3, 4, 10, 12, 13, 14, 19^	+ ^1, 3, 4, 10, 12, 13, 14, 19^				+ ^1, 4, 5, 14, 19^	+ ^1, 4, 5, 14, 19^			
UWR_735	*Escherichia coli*	+ ^1, 10,^ ^11, 14, 15, 19^	+ ^1, 10,^ ^11, 14, 15, 19^	+ ^1, 10,^ ^11, 14, 15, 19^			+ ^1, 11, 15, 19^	+ ^1, 11, 15, 19^	+ ^1, 11, 15, 19^		
UWR_736	*Klebsiella quasipneumoniae*	+ ^15^	+ ^15^	+ ^15^			+ ^1, 8, 11, 15, 19^	+ ^1, 8, 11, 15, 19^	+ ^1, 8, 11, 15, 19^		
UWR_737	*Micrococcus luteus*	+ ^1, 2, 12, 19, 20, 21, 23^	+ ^1, 2, 12, 19, 20, 21, 23^				+ ^1, 12, 19^	+ ^1, 12, 19^			
UWR_738	*Proteus mirabilis*	+ ^4, 5, 8, 9, 10, 11, 12, 13, 14,^ ^15, 16, 17, 18, 20, 21, 22, 23^	+ ^4, 5, 8, 9,^ ^10, 11, 12,^ ^13, 14, 15, 16, 17, 18, 20, 21, 22, 23^	+ ^4, 5, 8, 9,^ ^10, 11, 12, 13, 14, 15, 16,^ ^17, 18, 20, 21, 22, 23^			+ ^2, 3, 4, 5, 8, 9, 10, 11, 12,^ ^13, 14, 15,^ ^16, 17, 18,^ ^19, 20, 21, 22,^ ^23^	+ ^2, 3, 4, 5, 8, 9, 10, 11, 12,^ ^13, 14, 15, 16, 17, 18,^ ^19, 20, 21, 22,^ ^23^	+ ^2, 3, 4, 5, 8, 9, 10, 11, 12, 13, 14, 15, 16, 17, 18, 19, 20,^ ^21, 22, 23^		
UWR_739	*Pseudomonas aeruginosa*	+ ^6, 20, 21^	+ ^6, 20, 21^	+ ^6, 20, 21^			+ ^6, 7, 17, 18, 20, 21, 22^	+ ^6, 7, 17, 18, 20, 21, 22^	+ ^6, 7, 17, 18, 20, 21, 22^		
UWR_740	*Pseudomonas monteilii*						+ ^4^	+ ^4^	+ ^4^		
UWR_741	*Serratia marcescens*	+ ^1, 23^	+ ^1, 23^	+ ^1, 23^	+ ^1, 23^			+ ^23^			
UWR_742	*Staphylococcus aureus*	+ ^3, 8, 14, 15^	+ ^3, 8, 14, 15^		+ ^3, 8, 14, 15^		+ ^13, 14^	+ ^13, 14^		+ ^13, 14^	
UWR_743		+ ^10, 13^	+ ^10, 13^		+ ^10, 13^		+ ^14, 19, 20, 21, 23^	+ ^14, 19, 20, 21, 23^		+ ^14, 19, 20, 21 23^	

^1^A (+) sign indicates that the fungi have been isolated from a specific site. Labelling of individual material collection sites: 1. face, 2. left temporal region, 3. mental region, 4. trachea, 5. right auricle, 6. right hand, 7. left hand, 8. lungs, 9. diaphragmatic pleura, 10. liver, 11. liver parenchyma, 12. porta hepatis, 13. intestinal vasculature, 14. chest, 15. abdominal region, 16. back, 17. thigh and right knee, 18. thigh and left knee, 19. right calf first colony type, 20. right calf second colony type, 21. right calf third colony type, 22. left calf, 23. feet

The percentage distribution of bacterial isolates is shown in Fig. 2D, E, F. In the case of the necrobacteriome, the domination of *Proteus mirabilis* (27.94%), *Pseudomonas aeruginosa* (12.15%), and *Staphylococcus aureus* (10.93%) was observed (Fig. 2D). In contrast, the distribution of species within the thanatobacteriome showed a slightly different pattern – *P. mirabilis* remained the dominant species (38.89%), followed by *Enterobacter hormaechei* (13.89%), *S. aureus* (11.11%), and *Enterococcus faecalis* (9.26%) (Fig. 2E). Overall, the same species also dominated (Fig. 2F). For each sampling location, bacterial growth was assessed on five different culture media. Species occurrence was recorded as presence or absence on each medium, and the total number of positive isolations was used to evaluate their distribution and frequency.

### Fungal drug sensitivity

**Fig. 3:**
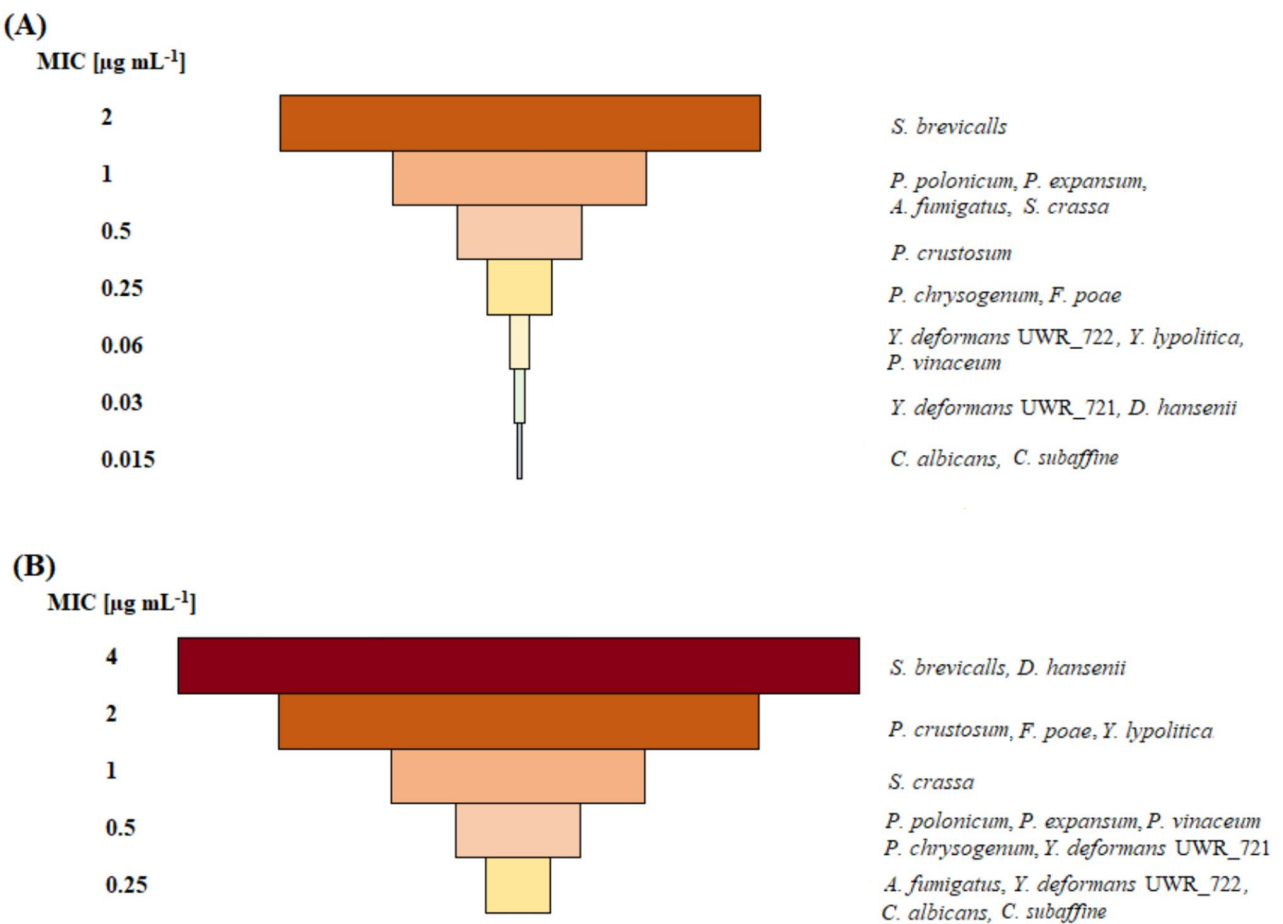
MIC values of voriconazole (**A**) and amphotericin B (**B**) plotted for tested strains

For the drug-sensitivity test, isolates capable of growth at 37 °C were chosen and these included: *C.* *albicans, Y. deformans* 110 and 112*, Y. lypolitica, D. hansenii, P. polonicum, P. expansum, P. chrysogenum, P. vinaceum, P. crustosum, A. fumigatus, S. brevicaulis, S. crassa, C. subaffine* and *F. poae*. The highest MIC (Minimum Inhibitory Concentration—the lowest concentration of the drug that completely inhibits the visible growth of the microorganism) value of voriconazole (VRZ) was obtained for *S. brevicaulis* (2 µg mL^−1^). *P. polonicum*, *P. expansum*, *A.* *fumigatus* and *S.* *crassa* growth was completely inhibited by 1 µg mL^−1^ of VRZ, followed by 0.5 µg mL^−1^ for *P. crustosum*. Voriconazole concentrations at a range of 0.25–0.06 µg mL^−1^ inhibited the growth of *P. chrysogenum*, *F. poae*, *Y.* *deformans* UWR_722, *Y. lypolitica* and *P. vinaceum*. The highest susceptibility to voriconazole was shown by *Y.* *deformans* UWR_721, *D. hansenii* (MIC of 0.03 µg mL^−1^), as well as *C. albicans* and *C. subaffine* (MIC of 0.015 µg mL^−1^; Fig. 3).

The highest MIC values of amphotericin B (AMB) were observed for *S. brevicaulis* and *D. hansenii* (4 µg mL^−1^) as well as *P. crustosum*, *F. poae* and *Y. lypolitica* (2 µg mL^−1^). Amphotericin B at 1 µg mL^−1^ completely inhibited the growth of *S. crassa*. *P. polonicum*, *P. expansum*, *P. chrysogenum*, *P. vinaceum* and *Y.* *deformans* UWR_721 growths were inhibited by AMB at 0.5 µg mL^−1^. The lowest resistance to AMB was shown by *A.* *fumigatus*, *Y.* *deformans* UWR_722, *C. albicans* and *C. subaffine*, with an MIC value of 0.25 µg mL^−1^ (Fig. 3).

### Bacterial drug sensitivity

Both *E. faecalis* and *Enterococcus faecium* strains were susceptible to ampicillin, high-dose aminoglycosides, tigecycline, vancomycin, and fluoroquinolones. *E. faecalis* showed intermediate susceptibility to imipenem, whereas *E. faecium* was resistant. In the *S. aureus* strain, the MLSB (macrolide-lincosamide-streptogramin B resistance) mechanism and methicillin resistance (MRSA – methicillin-resistant *Staphylococcus aureus* phenotype) were not detected, and this strain was also susceptible to the other antibiotics used in the test (Table 6).

**Table 6:** Sensitivity of bacteria to drugs

Isolate number	Species	Drug susceptible^1^	Intermediate susceptibility	Drug resistance
UWR_728	*Aeromonas hydrophila*	CAZ^2^, FEP, CIP, SXT		
UWR_729	*Bacillus tequilensis*	ERM, CLD, CIP, VA, IMP		
UWR_730	*Clostridioides difficile*	MTZ, VA		
UWR_731	*Corynebacterium amycolatum*	CIP, CLD, VA		
UWR_732	*Enterobacter hormaechei*	GEN, AK, AMC, FEP, CXM, TZP, IMP, AMP, SXT	CAZ, CIP	AMC, CXM
UWR_733	*Enterococcus faecium*	AM, TGC, GE, CIP,VA		IMP
UWR_734	*Enterococcus faecalis*	AM, TGC, GE,CIP,VA	IMP	
UWR_735	*Escherichia coli*	CIP, GEN, AK, AMC, FEP, CAZ, TZP, IMP, AMP, SXT		CXM
UWR_736	*Klebsiella quasipneumoniae*	CIP, GEN, AK, AMC, FEP, CAZ, CXM, TZP, IMP, AMP, SXT		
UWR_737	*Micrococcus luteus*	FOX, ERM, CLD, SXT, CIP, GEN, AK, AMC, FEP, CAZ, CXM, TZP, IMP, AMP, MTZ,VA		
UWR_738	*Proteus mirabilis*	CIP, GEN, AK, AMC, FEP, CAZ, CXM, TZP, IMP, AMP, SXT		
UWR_739	*Pseudomonas aeruginosa*	AK	CAZ, CIP, TZP, IMP	
UWR_740	*Pseudomonas monteilii*	AK	CAZ, CIP, TZP, IMP	
UWR_741	*Serratia marcescens*	CIP, GEN, AK, AMC, FEP, CAZ, CXM, TZP, IMP, AMP, SXT		
UWR_742	*Staphylococcus aureus*	FOX, ERM, CLD, SXT, CIP, GEN, AK, AMC, FEP, CAZ, CXM, TGC, TZP, IMP, AMP, MTZ, VA		
UWR_743	*Staphylococcus aureus*	FOX, ERM, CLD, SXT, CIP, GEN, AK, AMC, FEP, CAZ, CXM, TGC, TZP, IMP, AMP, MTZ, VA		

^1^selection of antibiotics in accordance with EUCAST, ^2^full name of antibiotics in the Table 1

Among Gram-negative *Enterobacteriales*, strains *Klebsiella quasipneumoniae*, *P. mirabilis* and *E. coli* were susceptible to all used antibiotics except for cefuroxime in the case of the *E. coli* strain. *E. hormaechei* showed resistance to amoxicillin with clavulanic acid and cefuroxime, and moderate susceptibility to ceftazidime and ciprofloxacin. Non-fermenting bacilli *P. aeruginosa* and *P. monteilii* were susceptible to amikacin and exhibited intermediate susceptibility to piperacillin with tazobactam, as well as to imipenem, ceftazidime and ciprofloxacin. The remaining species were susceptible to all tested antibiotic*s* (Table 6).

## Discussion

### Microbial communites

During body decomposition, microbial communities undergo several major shifts characteristic of different stages of decomposition, and “early communities” are significantly diverse from “late communities”. Typically, a corpse undergoes five stages of decomposition: fresh, bloat, active decay, advanced decay and dry remains (DeBruyn and Hauther 2017; Javan et al. 2016). According to the autopsy report, the studied corpse showed skin discoloration, maceration of the integument, lamellar separation of flaccid, grayish epidermis, disintegration of the internal organs’ anatomical structures with progressive tissue maceration and imbibition with blood pigment. These findings are characteristic of active decay. Samples obtained during autopsy included swabs from skin and organs; thus both, the external and internal microbial communities were investigated. The vast majority of isolated bacteria represented natural human gut and skin microflora, mainly from the *Enterobacteriaceae* family, but staphylococci were also found. On the other hand, *Aeromonas hydrophila* was found on swabs from the chin and palm. This species is classified as a primary or secondary pathogen, commonly found in aquatic environments (Semwal et al. 2023). Although its presence on the corpse may point to ante-mortem infection, this pathogen could have colonized postmortem; ante-mortem infection cannot be confirmed, since the corpse had been found outdoors and most likely had contact with soil. The presence of soil microorganisms on the corpse in the active decay stage is expected since ruptured body coverings can allow microbial migration. The release of bodily fluids creates favorable conditions for bacterial colonization (Moreno et al. 2011). Apart from *A. hydrophila,* other soil-associated species were detected on the studied corpse, including *Pseudomonas* spp. and *Bacillus* spp. *P. aeruginosa* is of particular concern because it is not only a common environmental isolate, but also a highly pathogenic species capable of causing severe infections, including those involving bones and wounds. Owing to its numerous virulence factors and both intrinsic and acquired resistance to antibiotics, it represents a significant biosafety risk for personnel handling human remains (Qin et al. 2022). It is worth mentioning that representatives of the gut microflora were found in the majority of the samples. While their presence in internal organs such as the liver and intestines is expected, isolates of the *Enterobacteriaceae* family were also recovered from skin samples taken from the legs, palms, and head. This finding suggests advanced postmortem bacterial translocation from the gastrointestinal tract to more peripheral body sites (Gates et al. 2021). These observations are consistent with the forensic examination of the corpse, which indicated the advanced decay stage of decomposition.

## Mycological methods and culture conditions

Forensic mycology is currently gaining attention due to the rising potential of fungi in criminal contexts. As the role of fungal microtraces in forensic cases has been documented (Hawksworth and Wiltshire 2011), the general knowledge of the colonization of corpses by fungi and their role in cadaver decomposition needs expanding. The present work involved a comprehensive mycological analysis of samples obtained from the cadaver overgrown with fungal hyphae to a large extent and numerous fungal species were identified from organs and body surfaces. To obtain the broadest spectrum of fungal species, the analysis employed four different mycological media and three incubation temperatures. Potato Dextrose Agar (PDA) and Sabouraud Dextrose Agar (SDA) are commonly used for the isolation of fungi; however PDA is generally more suitable for environmental samples, while the latter is usually applied for clinical specimens (Ogórek et al. 2024; Scognamiglio et al. 2010). Since cadavers may be considered as representing both environmental and human-derived material, the use of these two culture media is justified to ensure comprehensive fungal recovery. Additionally, YPD medium with a reduced pH was employed. YPD generally favors the growth of yeast forms (Suchodolski et al. 2025), while the acidic environment typically inhibits mold development. However, filamentous fungi were also isolated in our study using this medium, suggesting their tolerance to low pH. This observation is not unexpected, as the pH of blood and muscle tissue decreases post-mortem due to processes such as glycolysis and phosphofructokinase activity (Wang et al. 2021), creating an environment conducive to the survival and proliferation of fungi capable of withstanding acidic conditions. Lastly, the modified Leeming and Notman agar medium was used. Although this medium is dedicated to the isolation of *Malassezia* spp., it is not selective and also supports the growth of molds and *Candida* spp. (Suzuki et al. 2022; Abdillah and Ranque 2021), which aligns with our observations. The use of three different incubation temperatures minimized the risk of overlooking fungi with narrow thermal growth preferences and broadened the overall scope of mycological analysis, as previously demonstrated (Ogórek et al. 2024). A low temperature (5 °C) favors the growth of psychrophilic species, whereas incubation at 37 °C enables the detection of species associated with the human body (Ogórek et al. 2024). However, the highest number of isolates was obtained at 25 °C, which is considered the optimal growth temperature for the majority of fungal species (Borzęcka et al. 2021).

## Fungal community composition and succession

Mycological analysis of various sampling sites revealed that external body parts harbored a more diverse fungal community than internal areas. However, a clear dominance of *Penicillium* spp. was observed in both sample types. Most of the isolated *Penicillium* species (including the most abundant *P. polonicum* and *P. commune*) are common soil fungi (Samson et al. 2019); therefore, their presence is expected on a corpse that has been in prolonged contact with soil. The dissemination of fungi to body locations distant from the original colonization site has also been documented by Di Piazza et al. (2018), who reported an extensive overgrowth of *P. polonicum* and *Penicillium rubrum* covering 40% of the body surface. Alongside filamentous fungi of the genus *Penicillium*, frequent isolation of yeasts *D. hansenii* and *Y. lipolytica* from external and internal body parts was observed in our studies. The persistence, growth and diversity of microorganisms on a substrate—in this case a human corpse—are determined by environmental conditions (e.g., temperature, nutrient availability, water activity) and by fungal adaptations that enable survival and growth under such circumstances. In the present case, the deceased body was deposited in an open gazebo in early spring (with air temperature reaching −3 °C at night), and subsequently stored for 17 days at 4 °C prior to sampling. Given these environmental conditions and postmortem storage parameters, the presence of numerous psychrophilic or psychrotrophic species among the isolates is consistent with expectations. These include those identified by us: *P.* *expansum*, *P.* *crustosum*, *P. chrysogenum*, *P. polonicum*, *Penicillium solitum*, *Pseudogymnoascus pannorum*, *Helicostylum pulchrum*, and *Mucor racemosus* (Sato et al. 2013; Zhou et al. 2018; Hashem et al. 2022; Hassan et al. 2016; Al-Askar et al. 2021; Goncalves et al. 2013). As desiccation of tissues is a common feature of the decomposition process, fungal taxa capable of tolerating low water activity—such as members of the genus *Penicillium*—may persist and proliferate on cadavers (Piepenbring et al. 2025). Although a considerable number of fungal species have previously been reported from human remains (Piepenbring et al. 2025), several taxa recovered in our study represent new records in this context. These include *Botryotrichum domesticum* (a relatively novel species, found in indoor environments), *C. subaffine* (an endophytic fungus), *Penicillium allii* (a garlic pathogen), *S. crassa* (previously found in caves), *Sscopulariopsis* *fusca* (found in soda soils) and *Y. deformans* (Schultes et al. 2019; Dwibedi et al. 2023; Valdez et al. 2006; Zhang et al. 2017; Grum-Grzhimaylo et al. 2016).

## Functional potential (enzymes, decomposition)

The role of fungi in the decomposition of cadavers remains poorly characterized, as bacteria and insects are generally regarded as the primary agents in these processes (Hyde et al. 2013; Maisonhaute and Forbes 2023). Nevertheless, fungi are natural decomposers, capable of secreting a wide range of extracellular enzymes that facilitate the degradation of complex organic substrates (Kango et al. 2019). Proteinases, lipases, and amylases are considered the most relevant enzymes in cadaver decomposition, and multiple fungal species, including those isolated in our studies, have been reported to produce them. Proteolytic activity has been reported for *A. fumigatus* and *P. chrysogenum* (Yike 2011; Haq et al. 2006)*.* Lipase production is widely documented in *Yarrowia* sp., *A. fumigatus* and *P. chrysogenum* (Kumar et al. 2023), while *P. polonicum*—one of the most frequent isolates in our studies—has also been shown to produce lipases (Carvalho et al. 2024). The best fungal producers of α-amylases include *A. fumigatus*, *P. expansum* and *P. chrysogenum* (Farooq et al. 2021).

## Antimicrobial resistance and pathogenicity

It is important to emphasize that microorganisms associated with decomposing human remains may include species with pathogenic potential. Therefore, individuals who come into contact with corpses or environments contaminated by corpses should exercise particular caution. In the event of potential infection, knowledge of available treatment options is critical; therefore, antimicrobial susceptibility testing constitutes a crucial role. In this study, the majority of isolated bacterial strains were susceptible to antibiotics recommended by EUCAST guidelines (EUCAST 2025), suggesting a successful treatment in the case of an infection. In contrast, *E. hormaechei* exhibited resistance or intermediate susceptibility to antibiotics. This environmental species is also a frequent opportunistic human pathogen with well-documented mechanisms of resistance to multiple antimicrobial agents (Yeh et al. 2022).

Among the fungal isolates, those capable of growth at human body temperature were selected for susceptibility testing, as the ability to survive at 37 °C, and even within the fever range (38–42 °C), is a key factor for the development of systemic infections (van Burik and Magee 2001). Amphotericin B and voriconazole were selected for antifungal testing as ones of the most frequently used in the treatment of systemic fungal infections (Bouz and Doležal 2021). Defining a fungal strain as susceptible or resistant remains highly constrained due to the limited availability of standardized susceptibility data, which currently exist for only a small number of fungal species (EUCAST 2024). Of the species isolated in this study, *C. albicans* and *A. fumigatus* are the only ones for which clinical breakpoints for antifungal agents have been established (EUCAST 2025). Although both of them were classified as susceptible to amphotericin B and voriconazole., reduced susceptibility was observed for several other isolates. Notably, isolates of *D. hansenii* and *S. brevicaulis* exhibited the highest resistance to amphotericin B, which is consistent with previous reports for these species (Wagner et al. 2005; Cuenca-Estrella et al. 2003). The MICs of tested drugs for the remaining species were generally lower, indicating decreased resistance to amphotericin and voriconazole. Nevertheless, several of them have been described as opportunistic human pathogens, including *A. fumigatus*, *C. subaffine* or *P. chrysogenum* (Tekaia and Latgé 2005; Ahmed et al. 2016; Geltner et al. 2013).

Bacteria isolated from human corpses are predominantly saprophytic species, most of which are opportunistic strains capable of acting as etiological agents of disease under favorable conditions. Most often, these are “endogenous infections”—originating from the tissues and organs of the deceased, as well as exogenous infections—derived from the surrounding environment and the location where the body was found, known as putrefactive bacteria, which include bacteria of the genus *Proteus*, *Escherichia*, *Klebsiella*, *Clostridium* and also *Morganella*, and *Providencia* (Jangid et al. 2023).

## Forensic implications (PMI, environmental reconstruction, biosafety)

The findings of this study have several important implications for forensic science. First, the identification of specific microbial and fungal taxa associated with distinct anatomical regions and decomposition stages may aid in estimating the postmortem interval (PMI), particularly when traditional entomological or morphological indicators are unavailable (Metcalf et al. 2013; Speruda et al. 2022; Szleszkowski et al. 2022; Spychała et al. 2024). Identifying specific species associated with environmental conditions may enable the development of more refined PMI models tailored to regional conditions. Second, the presence of soil- and environment-derived microorganisms, including psychrotolerant and psychrophilic species, may provide valuable information about the location and conditions of body deposition, assisting in the reconstruction of the death scene (Metcalf et al. 2013; Spychała et al. 2025 unpublished). The study demonstrates the diversity and activity of fungi across different parts of the body during the active stage of decomposition, emphasizing their role as decomposers and potential bioindicators, and enhancing our understanding of their interaction with the environment. Third, the detection of drug-resistant bacterial and fungal strains highlights a potential epidemiological risk for forensic personnel, first responders, and others who may come into contact with decomposing remains (Deng et al. 2025). Identification of these resistant strains enables improved assessment of exposure risks and supports the implementation of evidence-based biosafety protocols, including appropriate personal protective equipment and hygiene measures. Overall, these findings highlight the necessity for stringent biosafety practices and further research into the transmission potential of postmortem microbiota.

## Data Availability

All data generated or analyzed during this study is included in this article.
